# Its Time-Table, Attendance, and Staff—III

**Published:** 1912-05-25

**Authors:** 


### THE IDEAL SCHOOL CLINIC.

### Its Time-table, Attendance, and Staff—III.

The work of the clinic cannot be carried on with the best and most successful results unless it is carried on in a systematic manner. While clockwork arrangements generally break down in actual practice, an attempt should be made to secure uniformity, to prevent economic waste and loss of energy. One of the first essentials is therefore a time-table which gives to every one connected with the institution a scale of regular duties which should not be deviated from. We submit the following as a specimen: to be modified to suit local needs:—

Monday: 8 to 9.—Medical and surgical cases. Treatment and examination.
1 to 2.—Orthopædic cases for treatment by exercises.
4.30 to 5.30.—Medical and surgical cases: Treatment.

Tuesday: 8 to 9.—Dental and medical cases. Treatment and examination.
1 to 2.—Skin cases: Treatment and examination.
4.30 to 5.—Medical and surgical cases: Treatment.

Wednesday: 8 to 9.—Surgical and eye cases: Treatment and examination.
1 to 2.—Orthopædic cases: Exercises.
4.30 to 5.—Medical and surgical cases: Treatment.

Thursday: 8 to 9.—Medical and surgical cases: Treatment and examination.
1 to 2.—Skin cases: Treatment and examination.
4.30 to 5.—Medical and surgical cases: Treatment.

Friday: 8 to 9.—Dental and medical cases: Treatment and examination.
1 to 2.—Orthopædic cases: Exercises.
4.30 to 5.—Medical and surgical cases: Treatment.

Saturday: 8 to 10.—Surgical cases: Operations.
8 to 10.—Eye cases.
10 to 11.30.—Medical and surgical cases: Treatment.

This table gives the attendance of the medical officers as follows: Monday, 8 to 9, physician and surgeon. Tuesday, 8 to 9, physician and dentist; 1 to 2, dermatologist. Wednesday, 8 to 9, surgeon and eye surgeon. Thursday, 8 to 9, physician and surgeon; 1 to 2, dermatologist. Friday, 8 to 9, physician and dentist. Saturday, 8 to 10, surgeon and eye surgeon. The nurse attends daily (except Saturdays) on week days during these hours and takes charge of the orthopædic cases requiring exercises, and from 4.30 to 5 of the surgical and medical cases requiring simple treatment. On Saturdays she attends to the latter class of cases from 10 to 11.30. Punctuality in attending the clinic must be one of the first considerations with the staff, and an attendance book must be kept in which every member of the staff enters his time of arrival at the clinic.

The number of nurses required for the ideal clinic with this time-table is at least four. If the clinic serves two districts it will have the services of two school nurses, who may be entrusted with the lighter duties, since all the work is done between school sessions. In that case allowance must be made for the time lost in going and coming from school to clinic. Two clinic nurses, whose duties are solely at the institution, must be engaged, and one of these should be trained to take charge of the orthopædic cases and superintend the drills and exercises. With a little attention and study these exercises are easily learned, and once the nurse is proficient in them she may safely be entrusted with the midday classes (as is the case in Germany) with an occasional supervisory visit from the surgeon.

Where punctuality is the rule there is no need to provide a refreshment stall in the waiting room, and to permit parents and children to bring food with them into the waiting room. Indeed everything should be done to discourage the habit of making the waiting room what so many hospital out-patient departments have become—a gathering place for the idle who come to enjoy the warmth and comfort and the chance of a bit of gossip. Immediate attendance where possible and the least amount of waiting in cases where summary examination and treatment cannot be secured, must be insisted upon. Where the clinic serves a number of schools it is convenient to take the scholars in batches from each school. Some authorities, however, prefer to divide the cases according to the character of the diseased condition. Both arrangements have their advantages, and individual inclination and local convenience will largely guide the staff in selecting either.

One of the most important officers of the clinic is the almoner or inquiry officer, who is usually a woman. On her rests the responsibility of finding what cases deserve free treatment. She makes out the patients' cards of admission or readmission, keeps a detailed register of cases seen and treated, and furnishes accounts to the secretary or to whomsoever is entrusted with the task of getting payment from the parents. Her position is therefore a responsible and delicate one, and it is advisable that she should be thoroughly acquainted with the condition of the population which the clinic serves, and that she should be in constant touch with teachers, medical staff, and nurses. Her attendance is required at the clinic whenever it is open—that is during the hours preceding the school session; she should therefore arrange her own time-table in such a way that she has an opportunity during the sessions themselves for her home visits, though much of her inquiry work may advantageously be done at the clinic itself, or at the schools. The clinic dispenser will not have a great deal to do, especially in clinics where stock mixtures are kept. The dispensary may therefore be entrusted to the nurse who assists the physician, if she has the necessary pharmacist's certificate. Otherwise a qualified dispenser must be engaged to attend at stated times and make up the prescriptions. Where the clinic serves a large number of schools a separate dispenser is nearly always required.

Thus the ideal clinic does not demand a very large staff nor very elaborate schemes of work. With a proper time-table, strictly adhered to, and proper co-operation between the workers in the various departments, all the work may be successfully coped with in the minimum amount of time, while the attendance of the children at the schools is not interfered with.

